# From Affective Arrangements to Affective Milieus

**DOI:** 10.3389/fpsyg.2020.611827

**Published:** 2021-01-25

**Authors:** Paul Schuetze

**Affiliations:** Institute of Cognitive Science, University of Osnabrück, Osnabrück, Germany

**Keywords:** situated affectivity, affect, affective arrangements, milieus, situatedness, social space, cultural affect

## Abstract

In this paper, I develop the concept of *affective milieus* by building on the recently established notion of *affective arrangements*. Affective arrangements bring together the more analytical research of situated affectivity with affect studies informed by cultural theory. As such, this concept takes a step past the usual synchronic understanding of situatedness toward an understanding of the social, dynamic, historical, and cultural situatedness of individuals in relation to situated affectivity. However, I argue that affective arrangements remain too narrow in their scope of analysis since their focus mainly lies on local, marked-off, and unique constellations of affect relations. They neglect the more mundane and day-to-day affect dynamics of social life. Hence, I introduce the notion of affective milieus, which brings to light the everyday, ubiquitous affective engagements of individuals with their socio-material surroundings. Affective milieus specifically call attention to how commonplace affect relations create territories in the social universe which form and mold individuals all the time. In that way, this paper apprehends and advances recent developments in the research on situated affectivity.

## Introduction

In recent years, the research on *situated affectivity* has led to the insight that affective phenomena should not only be analyzed in isolation, as marked-off individual mental states or happenings. Instead, researchers agree that these phenomena need to be addressed as being situated, as manifested in the interactions of agents and their surroundings (see e.g., Griffiths and Scarantino, 2009; Gallagher, 2013; Krueger, 2014; Colombetti and Krueger, 2015; Stephan and Walter, 2020). Advancing this endeavor, in the field of situated affectivity, the concept of *affective arrangements* was proposed as a theoretical tool to reveal that the intimate effects affectivity has on the interactional dynamics within socio-material settings (Slaby et al., 2019).^1^ This concept takes a step past the usual synchronic understanding of situatedness and goes beyond a focus on singular emotions, moods, existential feelings, or sentiments. It focuses on dynamic situatedness, on “local constellations of elements that give rise to specific relational domains of affecting and being affected” (Slaby et al., 2019, p. 5). Affective arrangements capture ensembles of persons, things, discourses, spaces, and behaviors in which *affect* is a unique modulator – they describe material-discursive formations orchestrated in compositions of particular *affect relations* (Slaby et al., 2019, p. 5). As such, they combine the socio-material situatedness of individuals and their affective relationality; and they emphasize that affect dynamics largely unfold between multiple actors in social domains of practice (Slaby, 2019b, p. 60). This makes possible an understanding of the social and cultural situatedness of individuals in relation to affectivity; and thereby, affective arrangements bring together the more analytical studies of affectivity and emotion (e.g., Griffiths and Scarantino, 2009; Stephan et al., 2014; Colombetti and Krueger, 2015), and affect studies informed by cultural theory (e.g., Gregg and Seigworth, 2010).

Within the current debate, this, of course, takes situated views of affectivity for granted (see e.g., Gallagher, 2013; Colombetti, 2018; Stephan and Walter, 2020); and basic assumptions from this field are presupposed, most importantly that “there is no pre-formed, independently existing individual that comes into a pre-formed, independently existing world[...]. Rather, it is the environment and the individual which together determine who and what they are” (Stephan and Walter, 2020, p. 15). Building on these assumptions, in the following, I survey the concept of affective arrangements in more detail, illustrating its core characteristics. However, I argue that affective arrangements remain too narrow. As I will elaborate in the Affective Arrangements section, by focusing on local and specific situations, they only capture special kinds of marked-off arrangements and address only very particular affect relations. Even though affective arrangements enable an understanding of the social situatedness of individuals in terms of affect relations in the first place, they neglect a societal and more large-scale view on situated affectivity. Thus, I take the theoretical concept of affective arrangements as an outset, and I apply its central ideas to the societal level. In doing so, I introduce the notion of *affective milieus*.^1^ Crucially, in contrast to affective arrangements, the concept of affective milieus is not attached to unique and local ensembles, but it brings to light acculturated and situated modes of being in general.^2^ It calls attention to a person’s habitualized affective engagements with her socio-material surroundings, how this relationality shapes her entire mode of being, not only in idiosyncratic and demarcated situations, and how this engagement manifests in particular spaces of the social world.

The first half of this paper will be concerned with laying out the conceptual framework of affective arrangements. I start with an introduction to the idea of *relational affect*, a central aspect of affective arrangements. Secondly, I introduce the concept of affective arrangements and analyze it in more detail. Then, I move beyond the concept of affective arrangements by pointing out its shortcomings while still upholding its main ideas. The second half of this paper develops the notion of affective milieus. Building upon the essential features of affective arrangements, I apply the key insights to a more societal and large-scale view. Finally, I illustrate the significance of affective milieus with a concrete example making clear the advantages this concept brings with it.

## Affective Arrangements

### Relational Affect

The notion of affective arrangements builds on an understanding of affect as a relational phenomenon, as not being restricted to individual agents, but as being a dynamic between bodies of various kinds (Slaby, 2019b, p. 61). The following provides a short introduction to this relational conception of affect, making way for a more detailed analysis of affective arrangements. The idea of relational affect takes a perspective on affectivity which focuses on situatedness and relationality, i.e., on the material and ideational relations unfolding “between […] ‘bodies’ whose potentialities and tendencies are thereby continuously modulated in mutual interplay” (Slaby et al., 2019, p. 4). To get a grip on this idea, take the following example: Suppose you are sitting at a restaurant table with some friends. You are loosely talking, arguing, and laughing while eating, drinking, and simply being there with each other. As is often the case in these situations, you may feel inclined to lean toward one side of the table and engage with this side more than the other, or you may only want to talk with the person sitting right across from you. Sometimes, it is even the case that the seating order already determines how the evening will go, how you will experience the atmosphere, how long you will want to stay, and how enjoyable the conversations will be. With any person leaving or joining the table, the whole situation can change; what was an intimate conversation may turn into shallow small talk, or what was a boring back and forth may suddenly become exciting. Even more subtle factors, like you having a drink in front of you or not, might affect how the evening evolves.

Intuitively, one will recognize all of these more or less subtle experiences and sensations. These are prime examples of the affect relations unfolding between social and material bodies. Yet, it will be difficult to put a finger on them, to specify what they truly are, because they are not graspable in terms of “clearly demarcated mental states” (Slaby, 2019b, p. 61). Rather, they are subtle changes in the relational dynamics between a person and her surroundings which influence how she experiences a situation and engages in it. These are the particularities which the notion of relational affect brings into focus.

In that sense, affect “is construed as a relational dynamic between individuals and in situations – a dynamic that is prior to individual experience, even, in a sense, prior to the individual subject as such” (Slaby, 2019b, p. 60). While “affectivity” denotes the general capacity of a person to be sensitive to, and affected by, what matters to her, “affect” characterizes the concrete relations in which a most basic form of affectivity substantiates. This relational idea of affect goes back to Baruch Spinoza’s complex metaphysical framework of substance monism (for a detailed discussion of this background, see Mühlhoff, 2018; Slaby and Mühlhoff, 2019). Without subscribing to the whole conceptual landscape of Spinoza, the essential point here is to recognize affect as a relational phenomenon which is constitutive of the individual subject (Seyfert, 2012; Mühlhoff, 2018). Affect, thus understood, might be viewed in terms of *relational affect dynamics* which express how a subject is situated in the world, i.e., in its social and physical surroundings (Mühlhoff, 2018, p. 20). This entails a “radically relational and dynamical understanding of individuals” which are grasped as “transiently stabilizing node[s] in an encompassing relational dynamic” (Slaby and Mühlhoff, 2019, p. 30). Individuals are constantly entangled in ways of affecting and being affected and they always have to be understood in terms of this relational fabric (Mühlhoff, 2018, p. 50). More specifically, “[t]he individual gets constituted processually … in a network of affective relatedness” (Mühlhoff, 2015, p. 1013). In that way, a focus on relational affect brings with it a developmental constructivist analysis of subjects (Slaby et al., 2019, p. 5). And so, the notion of affect puts emphasis on the base layer or the substructure on which the experiences, feelings, ultimately the individual subject itself is built upon (Åhäll and Gregory, 2015, p. 5). With this idea of relational affect in mind, the next section introduces and analyses the concept of *affective arrangements*.

### Affective Arrangements and Their Conceptual Background

Affective arrangements are first and foremost a theoretical tool to shine light onto local sociomaterial settings in which unique affect dynamics emerge and are continuously modified (Slaby et al., 2019, p. 3). In the following, I first provide an overview of the theoretical structure and the background of affective arrangements and then move on to a clarifying example.

Affective arrangements owe their name and their principal theoretical origin to the concept of *agencements* developed by Deleuze and Guattari (Deleuze and Guattari, 1987; Slaby et al., 2019).^3^ In their work, Deleuze and Guattari describe agencements as heterogeneous ensembles which consist of different artificial and natural components (Deleuze, 2006, p. 179). An agencement is a co-functioning unity which is defined in terms of the relations between its integrated elements; together these elements are laid out in an orchestrated, specific, and coherent whole in which they work together for a certain amount of time (Müller, 2015, p. 28). Yet, an agencement does not have an essence in and of itself, but it is entirely reliant on the relations of its elements, on the way, these elements are connected and work together coherently (Nail, 2017, p. 23). In an agencement, vastly different elements come together, and despite their difference, they portray a form of consistency, they create a unique identity and claim a territory in which the agencement persists (Wise, 2005, p. 77). In short, “an *agencement* is a fragmentary, open-textured formation: a concatenation of components that keep their distinctness” while still working together as a whole (Slaby et al., 2019, p. 6). To underpin this abstract idea with an intuitive picture, one may think of an agencement as a “dry-stone wall” (Deleuze and Guattari, 1994, p. 23). The individual elements, the stones, are not added and glued into a homogenous whole, rather they retain their individuality while still being part of a unity, a heterogeneous arrangement which works together as a whole, as a dry-stone wall.

However, since agencements only form the theoretical basis for the concept of affective arrangements, there are still differences. As the name already implies, affective arrangements put particular focus on affect relations, or more specifically, relational affect is their very basis. Going back to the dry-stone wall, one may say that affective arrangements are exactly that, fragmentary formations which form an orchestrated whole in virtue of their relatedness (Slaby, 2019a, p. 110). And, crucially, the relations between the elements are affect relations, i.e., affect is the glue which holds the stone wall together and prevents the stones from falling left and right. In that way, affect relations are the core of affective arrangements, they connect all elements within an arrangement, such that the arrangement becomes a unity demarcated from its surroundings (Slaby et al., 2019, p. 6). Following the concept of Deleuze’s and Guattari’s agencements, we can thus say that affective arrangements are ensembles of “persons, things, artifacts, spaces, discourses, behaviors, expressions, or other materials that coalesce into a coordinated formation of mutual affecting and being-affected” (Slaby, 2019a, p. 109).

Another defining precursor concept to affective arrangements is Foucault’s *dispositif* (Foucault, 1980; Slaby, 2019a, p. 109). Just like an agencement, the dispositif denotes a heterogenous ensemble consisting of various elements, such as discourses, institutions, laws, scientific statements, and philosophical and moral propositions, which are connected *via* their relations (Foucault, 1980, p. 194). And similarly, a dispositif describes the network of relations between the various elements. But other than agencements, a dispositif specifically highlights the social and political power structures that come with it (Seyfert, 2012, pp. 33–34). In that way, the notion of a dispositif captures the “strategies of relations of forces supporting, and supported by, types of knowledge,” (Foucault, 1980, p. 196) and as such it frames the setting in which certain things can be said, whereas others cannot – in which certain things can be conceived, whereas others cannot (Foucault, 1980, p. 194). What the idea of a dispositif adds to the texture of affective arrangements are the strategic power relations that manifest in ensembles of affect dynamics. Within an affective arrangement, the integrated elements take on specific roles, which are only partly due to their individuality, but which are largely the result of the relational framing of the respective formation (Slaby et al., 2019, p. 8). Moreover, a dispositif is defined by “a certain kind of genesis” (Foucault, 1980, p. 195). This means the network of relations of forces has a historicity – it always describes a particular way of becoming, of how it emerged and stabilized. Affective arrangements portray the same historicity. They are never just there, but “they emerge out of multiple formative trajectories, for example, histories of fine-tuning, of combining and recombining of components” (Slaby et al., 2019, p. 8). There is a genesis to affective arrangements manifested in the histories of the affect relations between its components, in habituated ways of affecting and being affected, and in the acculturation of rules, discourses, spaces, expressions, and other materials.

Summarizing the above, we may adhere that affective arrangements are heterogenous ensembles of natural and artificial elements, in which local patterns of affect dynamics form a unique affective texture. Such idiosyncratic formations are held together by specific affect relations; they prompt new affect dynamics, but also modify and guide them. Integrated individuals are subject to mechanistic relations, as they take on affective roles and acculturate modes of being, and by processes of habituation they become part of a functioning whole orchestrated by affect relations (Slaby, 2019a, p. 116).

To provide a clarifying example, consider a family gathering at Christmas. Parents, grandparents, children, aunts and uncles, cousins, and other relatives meet for their annual Christmas dinner. There are the classic tree, the candles, the Christmas smells, the typical food, and some presents on the side. All of this takes place at the same location each year, in the grandparents’ house. Importantly, this recurring event creates the same overall affective atmosphere: the feeling of Christmas. It is a historically grown tradition, which the family members have acculturated, and each new member, such as a new partner or a newborn child, is readily integrated. This illustrates the performative open-endedness of affective arrangements (Slaby, 2019a, p. 110). They are not pre-determined and rigid constellations, rather they possess a dynamic openness, in the sense that affective arrangements are “capable of expanding into their surroundings by incorporating new elements” (Slaby, 2019a, p. 110). Within the Christmas dinner there are natural and artificial elements integrated, and they come together to form a unity, a functioning whole. Each component, be it family member, tree, or present, retains its individuality, but takes on a role and becomes part of a network of relations creating the Christmas dinner. Much the same as a dry-stone wall, the Christmas dinner is a heterogenous ensemble consisting of distinct components which nevertheless cohere and create a unity held together by affect relations.

As the dinner carries on, some of the family will still be eating while the kids might already be finished and have left the table to play. By then, others will be in the kitchen, washing the dishes and preparing dessert. There are various interactions taking place simultaneously at different locations: the usual talk at the table, the more private conversation in the kitchen and the untamed play of the kids. While the overall pattern of the arrangement persists, it is constantly changing and transforming (Slaby, 2019a, p. 111). The different family members all have slightly different experiences, depending on their point of view and the people they are engaged with. Each member takes on a different role, which they have habituated over the years before, and which is strategically placed within the overall ensemble. One overall affective atmosphere has different yet similar segments, depending on the different affective interactions and relations. And all of this depends not only on the synchronic happenings, but also on the multi-track historicity of the affective arrangement, namely on the particular family history, traditions, and relationships, but also on “gender roles, cultural habits and commonsense behavioral expectations” (Slaby et al., 2019, p. 7). It is exactly this, the unity despite the situational diversity, the uniqueness of exactly this arrangement emerging from particular histories which come together, and the dynamic stabilization by processes of relational co-constitution, which is captured by an affective arrangement (Slaby, 2018a, pp. 209–210; Slaby et al., 2019, p. 7).

### Going Beyond Affective Arrangements

Having clarified the theoretical background, I now focus on aspects of affective arrangements which provide a starting point for introducing the larger-scale concept of affective milieus. An important point concerns the way individuals are seamlessly integrated in affective arrangements, how they attach to and are influenced by local affect-generating and co-constituting set-ups (Slaby, 2018a, p. 210; Slaby et al., 2019, p. 7). Most of the time, individuals automatically fit into the arrangement, they appropriately engage in the various interactions and become part of the whole by conforming to the overall pattern. Consider the Christmas dinner: Every family member behaves and feels according to the Christmas-like structure, according to the particular interaction partners (e.g., children or grandparents) and according to their specific location (e.g., kitchen or dinner table). In that way, even though there usually is no strongly felt pressure to abide to particular norms, each individual is integrated and acts according to a role (e.g., von Maur, 2018, p. 100). This means that mostly without noticing, without force, and mainly without actively being restricted in their individuality, all individuals being part of an affective arrangement behave and experience according to a role. In this way, the perspective of affective arrangements reveals the manner in which “subtle forms of a reciprocal affective interplay” produce and enforce entire modes of being (Slaby et al., 2019, p. 7). With reference to Foucault’s dispositif, this highlights the subliminal, yet influential, power relations which are at play in these networks of affecting and being affected.

Nonetheless, there are contrary situations in which the structures within an affective arrangement do not remain opaque, and the modulating plays of power are strongly felt. In the dinner example, suppose that one person at the table might start to make questionable jokes and comments. Commonly, there is an implicit rule to ignore these comments and not to make a big deal out of it for the sake of peace, so to speak. However, other guests might not want to let these comments be expressed unnoticed. For them, the affective arrangement is in tension with personal commitments, their roles within the overall formation deeply conflict with their individual identity. In those situations, norms of interaction are strongly felt, they appear at the surface and individuals are no longer seamlessly integrated. They notice how the situation binds them to a particular behavior, yet they want to act against it. Breaking one of these tacit norms in such situations requires effort, for it interrupts the fluidity of the situation and often causes irritation. The background nature of the affective arrangement will get lost and the affective atmosphere will possibly change. Here, it is important to note that such instances are less common compared to the situations in which one does not notice the underlying structures. Often times, individuals are unaware of the affective arrangements they are in, and so, for the most part affective arrangements seamlessly integrate individuals. But, most importantly, such tense situations emphasize the underlying force of affective arrangements. The effort it costs to deviate from the implicit rules and from the appropriate behavior or feeling (e.g., not laughing at the inappropriate jokes) indicates the force with which an affective arrangement usually incorporates individuals.^4^ From such set-ups of modulating and constituting affective relationality emerge new modes of being – “the individual subject … is … a complex ‘product’ of the sustained modulation by affect-intensive social domains” (Slaby, 2016, p. 2). Going back to Foucault’s dispositif, this makes explicit how the relations within an affective arrangement are relations of power. There are norms, ideas, and rules concretely embodied in these relations (Slaby, 2016, p. 8), such that individuals within the arrangement are subject to these power dynamics merely by being integrated in the arrangement – often being unaware of it (Slaby et al., 2019, p. 5).

Another essential aspect of affective arrangements is that they do not appear just like that, but they are the result of congregating histories, they are historically grown. The Christmas dinner is not realized from one day to another, but it has some kind of a genesis. This includes cultural trajectories, such as gender roles, behavioral norms, and other material-discursive processes, as well as the family and individual history. When these various lines meet and intersect, their concurrence creates the Christmas dinner. Such a multi-track historicity makes affective arrangements “‘conservation devices’ in which histories of interaction and of collective habituation have become sedimented” (Slaby et al., 2019, p. 7). This means that affect dynamics within affective arrangements necessarily rely on processes of becoming and on devices of acculturation, bringing to life a sedimented past (Slaby et al., 2019, p. 9). This particular emphasis takes a step away from a synchronic conception of situatedness. Instead it moves toward a complex, temporal, and diachronic comprehension of situated affectivity. In this way, affective arrangements acknowledge and provide a grip on the subtle, powerful, and intimate influences of affectivity – on the affective and “ontogenic dynamics that are formative of subjects” (Slaby et al., 2019, p. 7).

However, as mentioned in the beginning, I argue that affective arrangements still remain too narrow. They are geared toward picking out local settings of affect relations with a focus on idiosyncrasy. Not every family dinner is an arrangement, but only the ones that stick, the ones with a historicity, with a distinctiveness which makes people resonate (Slaby et al., 2019, pp. 5, 8–9). This means that the focus lies on singular and exceptional formations, such as the Christmas dinner. In order for this to happen, particular trajectories have to intersect and affect dynamics have to stabilize, forming “a unique local patterning of relational affect, giving shape to a potentially idiosyncratic affective texture or formation inherent in a specific place at a time” (Slaby, 2019a, p. 116). But despite this emphasis on local restriction and idiosyncrasy, affective arrangements tell us that situated affect dynamics are diachronic processes of becoming, that they orient and modify subjects, and that they form their entire mode of being. Evidently, this is not restricted to unique situations or formations such as the Christmas dinner, rather it is an everyday mechanism, repeatedly recurring. This is where affective arrangements are too limited in their scope. Such ontogenic processes do not just remain within physically restricted or “cranky” circumstances (Slaby et al., 2019, p. 8), but they subsist, they are there all the time. This is why the next section introduces the concept of *affective milieus*, taking seriously a more large-scale and wholistic view on situated affectivity. As such, affective milieus focus on *everydayness*: They bring to light the power relations manifested in the affect dynamics of social life, and they reveal the affective formative processes subjects are exposed to and immersed in every day.

## Affective Milieus

### Affect Dynamics as Orientation Devices

The theoretical tools of affective arrangements make apparent the concrete ways in which subjects are constituted by day-to-day affect dynamics. In order to further illustrate these fundamental mechanisms I bring to mind insights from the field of *critical phenomenology*. These approaches help to bridge the gap between the more localized analysis of affect dynamics, i.e., affective arrangements, and a large-scale, societal level viewpoint, i.e., affective milieus. Without building on the whole conceptual landscape of critical phenomenology, I focus on the rationale that historical and social structures “play a constitutive role in shaping the meaning and manner of our experience” (Guenther, 2020, p. 12). Essentially, this brings with it a large-scale analysis of the “social structures that make our experience of the world possible and meaningful” (Guenther, 2020, p. 15). Although these approaches are not explicitly developed in connection to affectivity, they nicely translate to the affective realm; and even tough they usually focus on the contingency of our experiences, they also shine light on general processes of subjectification. Take, for instance, the work of Sara Ahmed in “*Queer Phenomenology*” (Ahmed, 2006). In her approach, Ahmed describes the implications of what it means to be *oriented* in the world. While this idea is not concretely developed in terms of affect, it, nonetheless, helps to show that affect relations are powerful orientation devices in everyday interactions and not only in marked-off arrangements. Therefore, this work makes the transition from affective arrangements to affective milieus genuinely explicit. Now, while some of these insights are already implicitly present in the concept of affective arrangements, Ahmed’s detailed visualizations help to make them concrete.

Then, what does it mean to be oriented in the world? A person’s orientation determines what is close to her, and what is distant. Metaphorically speaking, things that are close are in sight, and things that are distant are out of sight. Different orientations limit what a person can do, what she can experience and what she may think. Ahmed starts with the simple example of sitting at a desk. Sitting and facing the desk implies a certain orientation in this very moment. Things on the table are near and in reach, things in the background are out of sight and out of reach. Being oriented toward the desk brings into focus specific matters. For instance, the work on the desk is of primary importance while the background, such as the family sitting in the kitchen, is of less interest. In that sense, the orientation toward the desk shapes what a person experiences as close or distant, as important or negligible, as doable or unfeasible (Ahmed, 2006, pp. 25–65).

Although, the concept of orientation is rather abstract, and Ahmed devotes large parts of her book to it, for the current purpose, it suffices to connect this idea to the study of situated affectivity. The very physical access Ahmed provides can be abstracted and applied in a figurative manner. To give a simple example from the realm of affectivity consider affective atmospheres (e.g., Riedel, 2019). A bright and sunny winter day with blue skies affects people in an entirely different way than a stormy, gray, rainy, and cold winter day. Both days have a very distinct atmosphere which people are embedded and entrenched in. Depending on this atmosphere, people may be oriented in a specific way, some things may be in sight, while others may remain hidden. For instance, on the sunny and bright day people may feel inclined to go outside, do exercise, or clean their apartment. Whereas on the rainy and gray day, they may want to be lazy, stay inside, watch a movie, or read a book. As Ahmed says: “What is reachable is determined precisely by orientations that we have already taken. Some objects do not even become objects of perception…: they are ‘beyond the horizon’ of the body, and thus out of reach” (Ahmed, 2006, p. 55). This example illustrates how Ahmed’s generic concept of orientation can be understood in connection to affective phenomena.

Subsequent to these ordinary examples, Ahmed points out that the concept of orientations also applies on a more fundamental and sustained level. It is not only in some situations that orientations are influential, but a person’s very mode of being is constituted by habituated orientations. Take again the Christmas dinner mentioned above: During the dinner, there are different roles for each family member, and these roles come with a specific orientation and ability to navigate. The socio-material affect dynamics composing the dinner orient and align all individuals in a certain way. As a consequence, the family implements implicit orientations without noticing and without force. This is enforced by the whole family: Unwittingly, every action and every word facilitate these orientations. With a nod to Deleuze’s and Guattari’s agencements, we can say that in the Christmas dinner, ensembles of trajectories come together aligning people in a peculiar way. Yet, importantly, such ensembles do not just develop in the face of the moment, but they are acculturated over years. And so, the dinner comes about as an arrangement of historically interwoven lines subjecting and habituating individuals to a unique material-discursive structure, enforcing orientations which outlast the moment and stick with the individuals over time (Ahmed, 2006, pp. 79–92).

However, as already mentioned above, such processes of habituation are not only present in unique situations, such as the Christmas dinner. Rather, we are always subjected to lines shaping and directing our orientations – “persistent social structures influence our capacity to experience the world, not just in isolated instances but in a way that is deeply constitutive of who we are and how we make sense of things” (Guenther, 2020, p. 13). In terms of relational affect, this reveals that affect dynamics act upon individuals all the time, they do not only orient individuals in certain atmospheres, such as a sunny or rainy day, or in specific situations, such as the Christmas dinner.

In the context of racism, specifically of *racializing perception*, Al-Saji (2014) provides a concrete example of how these processes unfold. Without going into too much detail here, Al-Saji analyzes how sedimentation and habituation manifested in affect relations tacitly constitute our visual processes such that certain things “*cannot be seen otherwise*” (Al-Saji, 2014, p. 138). As she states, “I can see bodies as raced only because I cannot see them otherwise,” (Al-Saji, 2014, p. 139) and this is deeply rooted in the habituated structures of our vision, as well as in acculturated affect relations configuring this vision. Now, while Al-Saji focuses on racialized bodies and racializing perception, at its core, her account is similar to what Ahmed captures with orientations. And so, this translates into a more general claim: “What is ‘otherwise’ is not only occluded from vision, but also from feeling, imagination, and understanding” (Al-Saji, 2014, p. 141). By analyzing the structures of vision, Al-Saji shows us how “habituated and socialized affects” form individuals in general – even primal processes, such as perception, are fundamentally constituted by patterns of affect relations (Al-Saji, 2014, p. 140). In other words, just as “habits of seeing owe to a social, cultural, and historical field,” (Al-Saji, 2014, p. 138) entire modes of being are the product of historically sedimented patterns of affecting and being affected (see also von Maur, 2018, pp. 224–232).

In short, the above connection to critical phenomenology highlights that affect dynamics form individuals, not only within affective arrangements but, more importantly, also in day-to-day dealings. All the affect relations a person is exposed to come together as transformative patterns of affecting and being affected, whereby they permanently constitute the person and how she makes sense of things. This means that affect dynamics are fundamental mechanisms of acculturation enforcing particular modes of being: They diachronically form what individuals find within reach, what they can see, what they can feel, and what they can think or imagine. This diachronic formation of subjects cannot be captured within the concept of affective arrangements, which remains within the limits of analyzing localized and idiosyncratic ensembles. Here, the notion of *affective milieus* makes a start.

### From Affective Arrangements to Affective Milieus

Generally, affective milieus inherit the core features of affective arrangements. Affective milieus are forms of agencements: They are heterogenous formations of natural and artificial elements which are held together by affect dynamics, but which do not have an essence in themselves; rather they are defined only in terms of these relational dynamics (Nail, 2017). They have a multi-track historicity as various trajectories intersect within them, and so they function as conservation devices preserving, molding and generating affect relations. Affective arrangements are like agencements “not simply a happenstance collocation of people, materials, and actions” (Buchanan, 2015, p. 385), but “a *specific* tangle of relations of affecting and being affected” (Slaby et al., 2019, p. 6). In the same way, affective milieus are specific tangles of everyday affect relations. Affective milieus also share the relational forces and plays of power captured by a dispositif. They are material-discursive ensembles developing, blocking, enforcing, and stabilizing power relations which support and are supported by types of knowledge (Foucault, 1980, p. 196). As such, they manifest in a network of forces held up by particular affect dynamics, and thus they subject integrated individuals to distinct power relations. In that way, affective milieus share the core features of affective arrangements, such as the ones adopted from agencements and dispositifs.

The crucial difference to affective arrangements is that affective milieus do not describe locally marked-off situations or ensembles which stabilize once in a while, which need to resonate with individuals, or which lure them into their positions (Slaby et al., 2019, p. 9). In a sense, affective milieus are always there: They do not usually have an attracting character, but they are structures residing in social domains of practice. They are *societal and large-scale* formations, which subdivide social space in a way that individuals are seamlessly integrated simply by being there. In contrast to affective arrangements, affective milieus are not cranky or strange compositions, they are not something extraordinary, and they are not something purposeful (Buchanan, 2015, p. 385; Slaby et al., 2019, p. 8). Affective milieus describe the day-to-day affect dynamics individuals are immersed in; they capture commonplace affect relations and identify them as powerful orientation devices, “as … process[es] of domestication – of making some objects and not others available” (Ahmed, 2006, p. 117).

This means that detached from affective arrangement, the spatial openness and the everydayness of affective milieus put focus on the permanent subjectification effects of affect dynamics. Affect relations are at the heart of a “material-discursive subject constitution … [which] … is a matter of effective framing and re-molding of subjectivity and selfhood” (Slaby, 2016, p. 7). It is exactly this aspect which affective milieus take up, as they shine light on the impact of large-scale societal formations. Adopting the notion of affective milieus highlights that “the subject is an active, environmentally embedded, and affectively situated agent” (Piredda and Candiotto, 2019, p. 136). As we have seen above in the digression into critical phenomenology, subjects are not only shaped by processes in unique localized situations, such as affective arrangements. But, the entire subject is constantly changing and building itself through ways of affecting and being affected (Piredda and Candiotto, 2019, p. 139). In other words, “every past experience of being-in-relation … shapes and forms the present and future individual potential” of the subject (Mühlhoff, 2015, p. 1013).^5^ This fundamental embeddedness in social space is picked out by affective milieus, and it is the dimension which marks the major difference to affective arrangements. In other words, the concept of affective milieus allows us to take a step back and get a grip on the various locally unbound affect relations which form an individual. This perspective goes beyond a selective focus on work environments, public transports, sports games, shopping malls, and other local settings (cf. Slaby et al., 2019, p. 9). Rather, this new angle of view puts emphasis on the multifaceted and ubiquitous affect relations coming together in a subject.

To clarify the difference between affective milieus and affective arrangements, take the following example: Suppose a person going home after a demonstration. The concept of an affective arrangement captures the particular dynamics of the demonstration; but once the demonstration is over, once this specific formation dissolves and the person detaches from the arrangement, the notion of an affective arrangement loses its grip. While the person leaves behind the particular affective arrangement, this does not necessarily entail leaving behind all of the affect dynamics or the orientations that were present within the arrangement. Instead, particular significance relationships might still remain with her, and particular dynamics may transfer to other areas of her life as well. For instance, when meeting friends after having participated in a rally, the topics of discussion will likely evolve around similar subjects; or when making certain decisions, the just experienced orientations will still remain influential. In that way, the affect dynamics live on in the individual. And so, these dynamics function as ongoing orientation devices bringing some things into sight, while making others impossible to see.

Of course, this does not happen immediately, merely by going to a single demonstration. But individuals are subject to infinitely many affect dynamics, not all of which are parts of affective arrangements such as demonstrations, but which might just be parts of daily routines, interactions, or other processes. These affect dynamics all come together in the individual; they do not suddenly vanish, nor can the individual simply detach from them. They move the individual, they stick with them, they embed them in ensembles of affect dynamics and relations, and thus they make up their lifeworld. Such dynamics do not remain singular points of contact, but they are part of a whole – they are parts of formations in the material and social life of individuals. They come together as a network of affect relations, meshed together, always transforming, stabilizing, modulating, and producing ways of affecting and being affecting. Ultimately, these affect dynamics constitute the individual. Such locally spread everyday dynamics are neglected by affective arrangements, and they are revealed by affective milieus.

### Affective Milieus

As shown above, although affective milieus inherit the core features of affective arrangements, there are some key differences. We have already seen that affective milieus are forms of agencements and forms of dispositifs. However, in contrast to agencements as taken up by affective arrangements, affective milieus are not highly localized, idiosyncratic structures with a mechanistic function. Rather, they are social formations which are there all the time. They reside in day-to-day socio-material relations and in the daily affective interactions of individuals. In that regard, affective milieus share the characteristics of a dispositif, as they are manifested in the relations between various elements on a social scale. Yet, different from a dispositif, affective milieus are composed of affect relations which function as the glue holding the various elements together. Moreover, milieus do not always have a “major function at a given historical moment” (Foucault, 1980, p. 195), as Foucault points out regarding dispositifs. Affective milieus can be without a historical function or a specific purpose. In essence, they are formations in social space, which individuals are always already situated within.

I mentioned before that affective milieus are large-scale societal formations. This means that in contrast to affective arrangements, they describe enduring ensembles of natural and artificial elements which are not restricted to a local setting. Yet, just as an agencement, affective milieus create a territory (Wise, 2005, p. 78). The affect dynamics composing an affective milieu occupy a certain space, they demarcate an area in which the milieu persists. Individuals integrated in these particular affect dynamics inhabit this territory, they are embedded in it such that the individual and the socio-material environment mutually constitute each other (Slaby, 2018b, pp. 331–332). Importantly, the territories so created have to be understood as abstract formations within the social space, as spaces defined by particular ways of affecting and being affected within social domains. This means that affective milieus do not literally delimit a marked-off physical territory. Rather, they demarcate a space in the social world understood as “a multidimensional system of co-ordinates” (Bourdieu, 1985, p. 724). In that way, affective milieus share core features with the spatial idea of a *social group*. Just as members of a social group “have a specific affinity with one another because of their similar experience or way of life” (Young, 2004, p. 43), elements of an affective milieu are connected to each other by similar affect relations and modes of being. As Iris Marion Young describes, social groups “are not entities that exist apart from individuals, but neither are they merely arbitrary classifications of individuals” (Young, 2004, p. 44). Similarly, affective milieus do not exist apart from their elements and the network of relations between them. It follows that an affective milieu only exists in virtue of shared and interconnected social and material relations of bodies; a specific milieu is not always there, but it has a history of stabilizing dynamics. This also means that these dynamics constantly change. The elements and the affective milieu constitute each other – as the elements change, the milieu changes and as the milieu changes, the elements change. Just like social groups are “fluid” constellations as “they come into being and may fade away” (Young, 2004, p. 47), affective milieus are constantly changing and transforming ensembles. Of course, these are not rapid transformations, but they entail a longer lasting development – a process of domestication and habituation, making some socio-material bodies and not others available by changing the affect relations between bodies over time.

At this point, it is important to note that the current paper merely gets a grip on the formations of affective milieus. In a next step it needs to be analyzed how these structures can be transformed, how they are not merely conservation devices, but possibly also vehicles of change. In this regard, further research may provide promising contributions, explicating how affective milieus can be altered and how individuals can change patterns of affecting and being affected. In fact, existing research on the transformative impact, especially of affective practices already offers fruitful insights into these questions (e.g., von Maur, 2018, ch. 5; Candiotto, 2019; Piredda and Candiotto, 2019). Once more, this highlights the unique perspective that comes with an analysis of affect dynamics in regards to societal issues: By its very nature it already provides access to avenues of change and to perspectives of rearrangement. And so, the concept of affective milieus not only presents itself as a descriptive tool but also offers space for transformative beginnings.

Now, the affiliation with the concept of a social group together with the perspective of societal change emphasizes the scale of affective milieus. Namely, they demarcate networks of affect relations on a *societal level*. As such, the concept of affective milieus functions similarly to the one of a social group; it arranges the social space into different formations. However, it is important to stress once again that affective milieus are composed of heterogenous elements. They are restricted neither to social nor to material relations, but they combine both. The linkage to social groups merely illustrates the scale on which affective milieus operate. Just as there are different social groups and classes, there are different affective milieus coexisting. This comparison makes concrete that an affective milieu is essentially a societal scale formation, subdividing the social universe into different territories, manifested in locally unbound affect relations.

Naturally, there are no sharp, clear-cut distinctions or absolute breaks in the social world (Bourdieu, 1987, p. 13). Therefore, affective milieus share aspects of what Pierre Bourdieu describes in the context of *social classes*. Social classes are overlapping, bordering upon one another with gradual borders, just as the “boundaries of a cloud or a forest” (Bourdieu, 1987, p. 13). The boundaries of a social class “can thus be conceived of as lines or as imaginary planes, such that the density (of the trees or of the water vapor) is higher on the one side and lower on the other” (Bourdieu, 1987, p. 13). The boundaries of an affective milieu are exactly the same. Just as a person can be right in the dense center of a forest, she can be in the intense, strongly integrated part of an affective milieu; and just as she can be at the light edge of the forest, where the forest gradually meets the meadow, she can be at the less intense edge of an affective milieu, where one milieu meets and passes into another. Social classes structure the social space and so do affective milieus. Abstractly speaking, a person is assigned an area within “the social universe” in virtue of a multitude of variables that apply to her, and this location attributes her to a certain class (Bourdieu, 1987, p. 4). In a similar manner, a person is located in the territory of a certain affective milieu depending on the affect relations she is embedded in.

Affective milieus demarcate territories in social space. These territories are almost like habitats for the integrated elements, they are socio-material environment these elements live in. Crucially, these territories are demarcated by particular affect dynamics where certain trajectories intersect and where unique ways of affecting and being affected are at play. By their very nature and by being formations within the social universe, affective milieus are not rigid, but fluid structures which are always transforming; and they are not clear-cut unities but dynamically open ensembles which are marked-off from their surroundings by gradual borders. All of the integrated individuals are similarly oriented and share a similar horizon, depending on their place within the affective milieu. This gives rise to an ensemble of elements, almost like a collective involving “shared orientations toward and around objects … [which] … would be an effect of the repetition of this direction over time” (Ahmed, 2006, p. 118).

To clarify the above, let us take an example and concretely apply the idea of an affective milieu. Suppose the cluster of environmentally conscious people, or more broadly speaking the *eco dispositif* if you will. The concept of an affective milieu allows us to frame this formation in terms of situated affectivity and grasp this formation as an *affective eco milieu*. This means that we can delineate a territory in social space where very particular socio-material relations are at play. For instance, the eco milieu may comprise individuals who share the same concerns, such as how to reduce plastic or CO_2_ emissions, or who have similar subjects to discuss with family and friends, for instance, how to buy more sustainable products. This territory may also be characterized by particular groups and specific activities, for example, individuals may come together and share their interest in gardening or farming. Moreover, this milieu also includes material relations such as owning sustainable clothes or foods, which are bought for instance from wholefood shops. And it may even be manifested in different kinds of work, as individuals may want to be doing something good for the world by choosing a workplace that accords with their principles. In short, there are very particular socio-material dynamics which compose the eco milieu. At its center, this milieu is a dense formation knitted by unique ways of affecting and being affected, and it gradually fades toward its edges, where the affect relations are loose-knit, where they overlap and intersect with bordering milieus. Depending on how strongly an individual is integrated and involved in the respective dynamics, it is located in the dense center or the lighter edges.

On the one hand, this example indicates that different individuals can be situated in different, even contrasting affective milieus. This would result simply from being involved in different affect dynamics. In the next section, I will go into more detail regarding this issue. On the other hand, this example also shows that there is a sort of unity and connection among the individuals integrated in the same affective milieu, simply because they are arranged in a shared network of relations which brings them together. They are part of a heterogenous formation, in which each person has her own life while still moving around the same socio-material settings as the others. Importantly, this example pinpoints the difference between affective arrangements and affective milieus by highlighting that individuals can meet in the same arrangements while being in a different milieu. Take for instance the Christmas dinner and the eco milieu. In one affective arrangement, the Christmas dinner, there may be individuals who are embedded in vastly different affective milieus, for instance, the eco milieu vs. an opposing milieu. And so, in contrast to affective arrangements, affective milieus can be described by a rather broad range of generic features, such as people sharing concerns; people engaging in similar topics; people exchanging and discussing with others the subjects that affect them, in families or with friends, at work or in their sports group; people reading or hearing the news and reacting in certain ways; and people buying and consuming similar media and other goods. Of course, this is only a small number of the dynamics which make up an affective milieu. Yet, they are examples of the concrete affect relations constituting affective milieus.

## Outlook: Future Perspectives

To show the significance of the concept of affective milieus, I bring to mind the topic of climate change. As an exemplary instance of this topic, I want to focus on the public debate about the sustainability of cars. This example will purposefully be exaggerated and I am well aware that there are more subtle undertones which I deliberately pass over. Yet, with this hyperbolic juxtaposition, I hope to pointedly contour the issues at stake, and to specifically highlight the unique understanding that comes with the notion of affective milieus. On the one side of the exemplary debate about the sustainability of cars, environmental activists demand that owning and driving cars ought to be more expensive to meet the actual costs of emitting an excessive amount of CO_2_ through individual transport. This should be achieved, for example, by introducing carbon taxes that would make gas more expensive. The contrary position – the car lobbyist – usually stresses the cultural and practical value of cars in addition to important social unrests that might result from higher gas prices (see e.g., the Yellow Vests movement). These two positions strongly oppose each other and whenever there is something to be done in either direction, reactions of the opposing side are harsh.

Framing this in terms of affective milieus, it becomes clear why both sides oppose each other so strongly. Usually, the wish for cars to be more expensive comes from younger people, often people (e.g., students and young families) who live in cities, where living without a car is rather easy. Moving within their environment is dominated by public transport, bikes, short distances to the supermarkets, and most places are within reach. Cars are even perceived as a burden for them. The streets are occupied by parking spots stealing valuable space within the city. Cars are loud and dirty, and they are making life among them unpleasant. Cyclists and pedestrians encounter cars as dangerous objects, almost as living entities which anonymously pass by accompanied by an aura of discomfort and fear. There are very particular affective relations such people have and do not have in connection to cars. Hence, they can easily conceive of a life without a car, and they may even enjoy the idea of a car-free city. Additionally, these people are immersed in very peculiar affect dynamics: They engage in certain activities, they might, for example, seek to escape the city by attending a small garden; they usually meet like-minded people who navigate in similar settings, and share the same work or living situation; they only consume particular things, e.g., exclusively buying environmentally friendly clothes and organic food.

The opposing side is often represented by people who own, love, and need a car. As such, they use a car more frequently, for instance to get to work or because they live in more rural areas. Their world is characterized by driving a lot, by long distances and spending a lot of time in or near their cars. They see an aesthetic value in owning a car. And so, a car is not simply a car, but an object of desire. This object should have certain favorable and appealing characteristics. For instance, owing an SUV in a city has no practical value at all, but it brings with it a peculiar feeling. And so, the affect relations these people have with and around their cars are vastly different to the ones described before. And similarly, these people are involved in their very own affect dynamics: Consumption priorities are different, e.g., their car has a high personal value, it signals their social status, motivating them to hold and spend their money accordingly; their social contacts largely evolve around people who also own cars and can only be reached by car, or with whom they go on trips and vacation. In contrast to the other camp, their areas of life are shaped less by environmental concerns, i.e., the activities these people are engaged in are not so much focused on environmental friendliness. They might for instance carelessly do winter sports or fly to vacation destinations, the clothes and food they buy might not be sustainably produced.

Each of the two camps is situated in an affective milieu with its peculiar socio-material dynamics. The people in each milieu are oriented in very different directions (although not always in such a contrasting manner). Very particular things come into reach and become possible when being oriented around a world involving cars or around a world without cars. This also means that within such an affective milieu only a limited set of solutions comes into sight when approaching a problem. For either of the two camps, it requires a lot of effort to see and comprehend the ideas and thoughts of the other side. This is simply because such ideas and thoughts are not within reach from the affective milieu they themselves are situated in. Relating back to the Christmas dinner example, it requires work to not just let the ignorant jokes slide at the table. One needs to step out of given norms and break with one’s habituated mode of being. In a similar way, individuals within the affective milieu of “liberal car-related people” need to step out of their habituated being in order to bring other solutions into reach.

Relating this to the broader example of climate change, we can see how the study of situated affectivity can contribute to the analysis of such issues. The concept of an affective milieu makes this contribution concrete. It is not enough to present people with new data in order for them to change their behavior, or their way of life more generally. It is not even enough to present them with the concerns of other people. For a person to look beyond her affective milieu, she needs to be aware of the specific power relation she is embedded in. Relating back to the field of critical phenomenology, the goal then needs to be to create possibilities of reflecting and changing one’s relations with the world.

## Author Contributions

The author confirms sole responsibility for the conception, development and composition of this article.

### Conflict of Interest

The author declares that the research was conducted in the absence of any commercial or financial relationships that could be construed as a potential conflict of interest.

## Footnotes

^1^Since the concept presented in this paper, i.e. affective milieus, builds on affective arrangements, it is significantly different from the one of affective scaffolding discussed by Colombetti and Krueger, 2015. This is because they have a different analytical focus, for details on this difference see e.g. Slaby, 2016.

^2^Merleau-Ponty also talks about “affective milieus” (e.g., Merleau-Ponty, 1945/2012, p. 156). But, different from the meaning pursued in the current paper, for Merleau-Ponty, an affective milieu manifests around an individual’s body. For him, an affective milieu is “the sector of our experience that clearly has sense and reality only for us” (Merleau-Ponty, 1945/2012, p. 156). As such, an affective milieu denotes the surroundings which affectively matter to the individual; these affective relations bring into existence these surroundings for the individual body in the first place (Merleau-Ponty, 1945/2012, p. 140; see also Roald et al., 2018). Since I am building on the notion of affective arrangements, I do not take up Mearleau-Ponty in the following.

^3^By “mode of being” I touch upon Heideggerian terminology, i.e., being-in-the-world. By different “modes of being” I, therefore, refer to different modes of being-in-the-world (see Wheeler, 2018; Thonhauser, 2020).

^4^The most common translation, retained by Brian Massumi, of *agencement* would be *assemblage.* But, as this brings with it various semantic problems, I make use of the original term (see e.g., Phillips, 2006; Buchanan, 2015).

^5^One may think of situations in which individuals feel strongly restricted by the norms at play. Take for instance the gender norms present in a very traditional Christmas dinner (e.g., women preparing the food and men talking at the table). Despite the imperative objections one should have when encountering such norms, the essential point remains: an individual’s passive integration into the arrangement. Norms are not explicitly set up – no one openly states these gender norms before every Christmas dinner – rather they subliminally guide individuals.

^6^Here, it is important to note that subjects can actively change their interaction with the environment, they can partly change the ways in which they affect and are being affected. This way, affective practices have a vital transformative character (see Candiotto, 2019; Piredda and Candiotto, 2019). However, in the current paper, I cannot take up this implication as I employ a descriptive approach particularly focusing on the substantial influences of already existing affect relations (see also Slaby, 2016).
